# Autoimmunity to the Follicle-Stimulating Hormone Receptor (FSHR) and Luteinizing Hormone Receptor (LHR) in Polycystic Ovarian Syndrome

**DOI:** 10.3390/ijms222413667

**Published:** 2021-12-20

**Authors:** Hanna A. Schniewind, Lisa-Marie Sattler, Christoph W. Haudum, Julia Münzker, Waldemar B. Minich, Barbara Obermayer-Pietsch, Lutz Schomburg

**Affiliations:** 1 Institute for Experimental Endocrinology, Charité-Universitätsmedizin Berlin, Corporate Member of Freie Universität Berlin, Humboldt-Universität zu Berlin, and Berlin Institute of Health, 13353 Berlin, Germany; hannaschniewind@googlemail.com (H.A.S.); lisamarie.sattler@googlemail.com (L.-M.S.); Waldemar.minich@charite.de (W.B.M.); 2 Endocrinology Lab Platform, Department of Internal Medicine and Gynecology and Obstetrics, Medical University Graz, A-8036 Graz, Austria; christoph.haudum@medunigraz.at (C.W.H.); Julia.muenzker@gmail.com (J.M.)

**Keywords:** autoantibody, luciferase, testosterone, G-protein coupled receptor, fertility

## Abstract

Hyperandrogenemia and ovulatory dysfunction are hallmarks of polycystic ovary syndrome (PCOS), pointing to a deranged hypothalamus-pituitary-ovarian (HPO) axis. An autoimmune etiology of PCOS is suspected in a subset of patients due to the relatively high concordance of PCOS with common autoimmune diseases. For this reason, we tested the hypothesis that natural autoantibodies (aAb) to the follicle-stimulating hormone receptor (FSHR) or luteinizing hormone receptor (LHR) are prevalent in PCOS. To this end, new luminometric assays for quantifying aAb to the FSHR (FSHR-aAb) or LHR (LHR-aAb) were developed using full-length recombinant human receptors as fusion proteins with luciferase as reporter. Prevalence of FSHR-aAb and LHR-aAb was determined in serum samples from healthy controls and PCOS patients. Steroid hormone profiles were compared between patients with and without FSHR-aAb or LHR-aAb. Signal linearity and detection ranges were characterized and both methods passed basic performance quality checks. The analysis revealed a relatively low prevalence, with 4 out of 430 samples positive for FSHR-aAb in the control versus 11 out of 550 samples in the PCOS group, i.e., 0.9% versus 2.0%, respectively. Similarly, there were only 5 samples positive for LHR-aAb in the control versus 2 samples in the PCOS group, i.e., 1.2% versus 0.4%, respectively. Samples positive for FSHR-aAb displayed steroid hormones in the typical range of PCOS patients, whereas the two samples positive for LHR-aAb showed relatively elevated free testosterone in relation to total testosterone concentrations with unclear significance. We conclude that the FSHR and LHR constitute potential autoantigens in human subjects. However, the prevalence of specific autoantibodies to these receptors is relatively low, both in control subjects and in women with PCOS. It is therefore unlikely that autoimmunity to the LHR or FSHR constitutes a frequent cause of hyperandrogenemia or ovulatory dysfunction in PCOS.

## 1. Introduction

Polycystic ovarian syndrome (PCOS) constitutes a common endocrine disorder affecting approximately 8–13% of adult European women, with the prevalence varying widely among different populations throughout the world [1]. Disease onset can often be observed already in adolescence [2]. The definition of PCOS includes morphological along with endocrine and symptomatic parameters, and the disease is currently diagnosed according to the Rotterdam criteria based on hyperandrogenemia, oligo-/anovulatory infertility, and/or polycystic ovary morphology [3]. A diagnosis of PCOS is made when two or three of these criteria are met, i.e., women without polycystic ovary morphology can also be diagnosed as PCOS patients [4]. This inconsistency highlights the complexity of the syndrome, the lack of understanding of its etiology, and the urgent need for refined criteria and additional diagnostic parameters [5].

Several lines of research are currently being pursued in the search for novel informative parameters to help identify the disease early, support treatment, and stratify the patients. These include molecular analyses testing for associated gene variants and genetic alterations, metabolome and proteome analyses from different matrices, immunological approaches to identify alterations in cells of the immune system and circulating autoantibodies, or microbiome analyses [6,7,8,9]. Based on the currently available results, a purely genetic or environmental origin of PCOS is unlikely, and no single genetic biomarkers, environmental triggers, or common circulating disease-specific autoantibodies have been identified so far [7]. This notion again underlines the complexity of the disease, despite the observation that PCOS often runs in families. Several generations of women are sometimes affected, and even some PCOS-like features can also be observed in the men from these families, including typical body shape, hair loss pattern, and high androgen levels, suggesting some common evolutionary advantages from the disease [10]. A second characteristic of PCOS suggests another important factor in disease etiology, namely the immune system. The risk for and prevalence of PCOS is elevated in women suffering from an autoimmune disease, especially in patients with Hashimoto’s thyroiditis [11], Graves’ disease [12], osteoarthritis [13], type 1 diabetes mellitus [14], systemic lupus erythematosus [15], or psoriasis [16]. Autoantibodies to thyroperoxidase (TPO-aAb), for example, were found in 26.7–26.9% of women with PCOS compared to 6.6–8.3% of healthy women in case-control studies in Turkey and Germany [17,18]. An increased TPO-aAb prevalence of 10–31% is generally found in adult women with subfertility and in 17–33% of women with recurrent pregnancy loss [19]. These numbers are considerably higher than the prevalence of 9.1–9.9% in the general female population [20], and further support the hypothesis of a relevant autoimmune component in a subgroup of patients with PCOS [21,22].

The hypothalamus-pituitary-ovary (HPO) axis controls ovarian development and function including follicle recruitment, oocyte selection, regular cycling, and steroid hormone biosynthesis [23]. The rhythmicity of hypothalamic gonadotropin-releasing hormone (GnRH) release into the portal blood system is the central coordinator from brain for the synthesis and release of the pituitary gonadotropins. The GnRH-receptor (GnRHR) on gonadotropic cells of the anterior pituitary translates circulating GnRH released from the neuroendocrine hypothalamic cells into the secretion of follicular-stimulating hormone (FSH) and luteinizing hormone (LH). Variation in GnRH pulse frequency and amplitude allows fine tuning of FSH versus LH release and controls the relative amounts of the circulating gonadotropins, resulting in different and dynamically changing FSH and LH concentrations (the so-called LH/FSH ratio, which is elevated in PCOS) [24]. Gonadotropin-specific receptors at the major target organ, i.e., the ovary in women and the testes in men, belong to the family of G-protein coupled receptors (GPCR) and translate the FSH and LH signals into endocrine effects. LH-receptors (LHR) are mainly located on theca cells in the ovary or Leydig cells in the testes and affect steroid hormone biosynthesis [25]. FSH-receptors (FSHR) on granulosa cells in the ovary or Sertoli cells in the testes control the recruitment and maturation of gonadal stem cells and the processing and transport of steroid hormones [26].

Endocrine receptors of the GPCR family are established targets in autoimmune diseases, with the receptor for thyrotropin (TSHR) taking center stage in autoimmune thyroid diseases, particularly Graves’ disease, where activating autoantibodies not only serve as diagnostic parameters but also represent the cause and a major therapeutic target of the disease [27]. Both the anterior pituitary glycoprotein hormones (FSH, LH, and TSH) and their receptors (FSHR, LHR, and TSHR) share a high degree of homology and belong to the same ancestral families [28,29]. It is therefore conceivable that the FSHR and the LHR represent disease-relevant autoantigens in human subjects, as the TSHR does in Graves’ disease. Some case reports or small case-control studies support this notion in premature ovarian failure, using radiolabeled ligands in displacement experiments or FSH-binding inhibition assays to investigate the presence of potentially interfering autoantibodies [30,31,32]. However, the results have not been verified by larger analyses, none of the assays received a wider application, and no indications for autoantibodies interfering with FSH action were observed in male infertility [33]. Nevertheless, to test this appealing hypothesis more systematically, we developed and validated novel non-radioactive in vitro assays capable of quantifying autoantibodies to these two GPCR directly and evaluated the prevalence of FSHR-aAb and LHR-aAb in a large cohort of PCOS patients and controls.

## 2. Results

**Figure 1:**
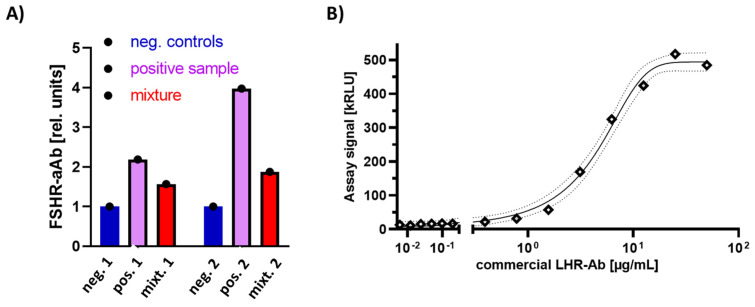
Performance test of the newly generated assays for quantifying autoantibodies to the FSHR and LHR. Recombinant full-length proteins of the human FSHR and LHR were expressed as fusion proteins with firefly luciferase in HEK293 cells and was used to detect and quantify receptor-binding antibodies. (**A**) Two samples positive for binding to the FSHR-luciferase fusion protein were identified in the serum samples analyzed; sample #1; slightly positive, and sample #2; highly positive. The positive samples were mixed (1:1, *v*/*v*) with negative control serum. The assay signal of the mixture was close to the expected arithmetic mean of the positive and negative samples, indicating the absence of matrix effects. (**B**) A commercial antiserum to human LHR was identified and tested in linear dilution with the newly generated LHR-aAb assay. The signals increased with antibody concentrations and indicated a dynamic and sufficiently wide measurement range.

Using the newly developed assays, a few positive serum samples were identified. Two serum samples positive for the FSHR-aAb were used in dilution experiments with negative samples to test for matrix effects. The mixtures of equal volumes of a negative sample (blue) with a positive sample (violet) yielded the expected mean signals (red) of both samples (Figure 1A). No deviation from this prediction was seen with the sample slightly positive (sample #1) or highly positive (#2). Unfortunately, no commercial antibody specifically binding the human FSHR was identified as an additional and independent positive control.

Specificity and linearity of the LHR-aAb assay was tested by dilution of a commercial antibody in binding buffer. A concentration-dependent decline of luciferase activity in the precipitate according to the applied amount of antiserum was observed (Figure 1B). Linearity of the signal extended over almost two orders of magnitude. The LHR-specific antibody showed no cross-reaction to the recombinant human FSHR.

**Figure 2:**
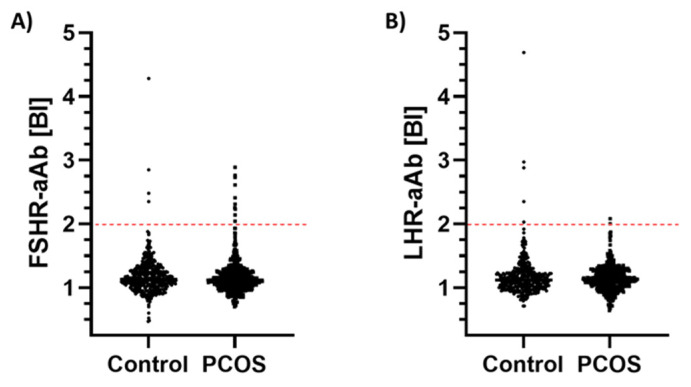
Comparison of control and PCOS samples for autoantibody prevalence to the human FSHR (FSHR-aAb) or LHR (LHR-aAb). A set of serum samples from control subjects (n = 430) and patients with PCOS (n = 550) was assessed for autoantibodies to (**A**) human FSHR (FSHR-aAb), and (**B**) human LHR (LHR-aAb), respectively. The signals obtained showed a skewed distribution with few samples only exceeding the threshold of positivity, i.e., showed signals above P75 + 3xIQR (criterion for extreme outliers), indicated by the red broken line. No obvious difference in prevalence of positive samples for FSHR-aAb or LHR-aAb was observed in controls versus PCOS patients.

The analysis of our cohort of serum samples for FSHR-aAb or LHR-aAb yielded a dataset of skewed signals and showed no normal distribution, indicative of positive samples exceeding the range that can be caused by technical noise alone. By applying the criterion for extreme outliers (P75 + 3xIQR), a small number of positive samples were identified with both assays. However, the signals were not exceedingly high. The prevalence of positive samples was similar in both cohorts, i.e., there was no obvious quantitative difference in autoimmunity against the FSHR and LHR in controls and PCOS patients (Figure 2). According to the cut-off criterion, there were four samples positive for FSHR-aAb in the control group (prevalence of 0.9%), and 11 positive samples in the PCOS cohort (prevalence of 2.0%) (Figure 2A). The prevalence for positivity to the LHR was similar, with five positive samples in the controls, and two in the PCOS group (prevalence of 1.2% and 0.4%, respectively) (Figure 2B). Notably, the positive samples did not overlap, i.e., the samples positive for LHR-aAb were from different subjects than those positive for FSHR-aAb.

**Figure 3:**
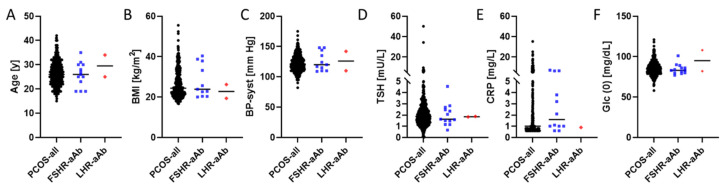
Comparison of anthropometric and clinical parameters of PCOS patients in relation to autoimmunity to the human FSHR or LHR. Patients were compared in terms of (**A**) age, (**B**) body mass index, and (**C**) systolic blood pressure, as well as (**D**) TSH, (**E**) C-reactive protein, and (**F**) fasted glucose concentrations. No pattern of consistent differences was observed in this analysis.

Anthropometric and clinical parameters were compared between the full cohort of PCOS patients and the autoantibody-positive patients (Figure 3). No obvious differences were observed for age (Figure 3A), BMI (Figure 3B), systolic blood pressure (Figure 3C), TSH (Figure 3D), CRP (Figure 3E), or fasted glucose concentrations (Figure 3F).

**Figure 4:**
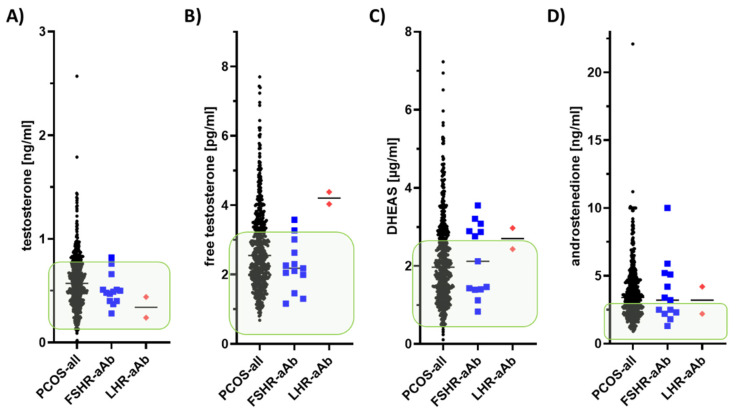
Comparison of steroid hormones in PCOS samples in relation to reference ranges and autoimmunity to the human FSHR or LHR. The serum samples from the PCOS patients were characterized for (**A**) total testosterone, (**B**) free testosterone, (**C**) dehydroepiandrosterone (DHEAS), and (**D**) androstenedione concentrations. The direct comparison of the full set of data from the PCOS samples (PCOS-all) to the reference ranges (indicated by green squares) and to the samples positive for autoantibodies to the human FSHR (FSHR-aAb) or LHR (LHR-aAb) is presented. No striking differences between within the groups of PCOS patients is observed, except for a seemingly elevated level of free testosterone in the two subjects positive for LHR-aAb.

Next, the steroid hormone patterns of the patients identified as positive for FSHR-aAb and LHR-aAb, respectively, were compared to the full cohort of PCOS patients (Figure 4). The concentrations of total testosterone (Figure 4A), free testosterone (Figure 4B), dehydroepiandrosterone (DHEAS) (Figure 4C), or androstenedione (Figure 4D) were similar between the three groups of patients. The only notable difference relates to free testosterone, where the two subjects positive for LHR-aAb seemed to display slightly elevated values.

**Figure 5:**
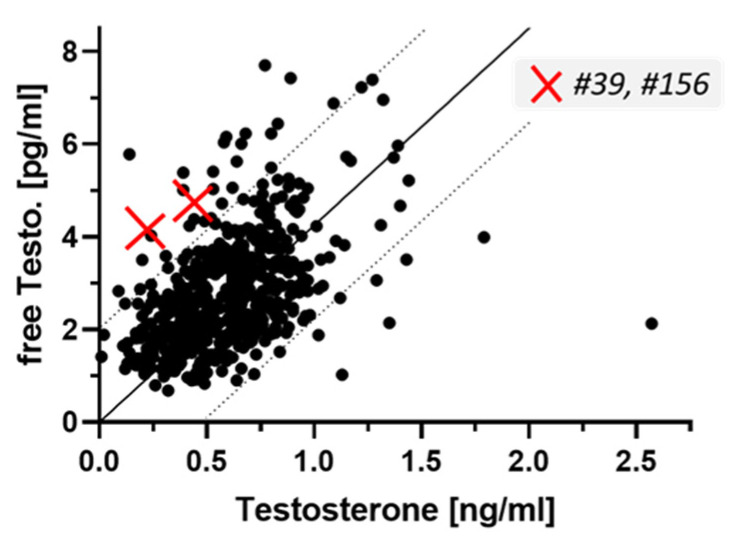
Testosterone and free testosterone concentrations in PCOS patients in relation to LHR-aAb. Two samples were identified as positive for autoantibodies to human LHR (samples #39 and #156, indicated by the red crosses in the xy-plot). In the full cohort of samples, the two steroid parameters show a strong linear correlation (Y = 4.251 × X, *p* < 0.0001). Testosterone and free testosterone concentrations of the two positive samples positive were outside the 95% and outside the 90% confidence interval, making an incidental finding not very likely.

A detailed analysis of the free and total testosterone concentrations indicated that the two positive samples for LHR-aAb (denoted as #39 and #156) were located outside the 90% confidence interval, and even outside the 95% confidence interval of the full group of patients (Figure 5). The latter is indicated as broken lines in the figure.

## 3. Discussion

This study describes two newly developed autoantibody assays for two human GPCR, a first characterization of their key performance parameters, and a parallel application of these assays to test their potential suitability to aid PCOS diagnosis and improve our understanding of the disease. The research was based on the assumption of an autoimmune etiology in a subgroup of PCOS patients, mainly due to the known elevated association of PCOS with other autoimmune diseases [34]. Both newly generated assays were found to be reliable in dilution experiments, and the performance of the LHR-aAb assay was successfully tested with a commercially available LHR antiserum. Unfortunately, no suitable commercial source was identified for specific antibodies to the FSHR. This limitation is a well-known and common problem when working with GPCR and looking for receptor-specific commercial antibodies [35,36,37]. The parallel development of both assays with the same strategies and tools supports the comparability of the measurements. The analysis of a relatively large cohort of PCOS patients and controls failed to reveal a particularly high prevalence of FSHR-aAb or LHR-aAb in the patients. A small number of positive samples were identified in both groups, indicating that both receptors can indeed be recognized as specific autoantigens in humans. However, the prevalence was low, the signals were not exceptionally high questioning pathophysiological relevance, and there was no obvious association with PCOS. From these data, we conclude that autoimmunity to the gonadotropin receptors is a rare finding that has no obvious relevance to routine diagnosis of PCOS. Whether the apparent deviation of testosterone concentrations in the two samples identified as positive for LHR-aAb is of pathophysiological relevance is doubtful and requires an independent verification in additional analytical studies, ideally of even greater size.

The reliability of the obviously disappointing main result of this study depends primarily on the quality of the novel analytical assays used and patient samples analyzed. The detection and reliable quantification of protein-specific autoantibodies is a critical and sensitive issue, and various methods and protocols have been developed [38,39]. From a technical point of view, the use of small antigenic peptides is most straightforward, as they can be commercially produced in the desired quantities and with high purity, immobilized on various surfaces or directly labeled with a range of different detection tags with good efficiency. This approach also allows a thorough characterization of the antigenic epitopes by screening a series of overlapping peptides covering the entire primary sequence of the antigen by high-throughput approaches, [40]. Alternatively, phage display libraries of sufficiently high complexity may be used [41]. However, several disadvantages can arise when using peptide-based approaches; (i) three dimensional or conformational epitopes consisting of distant residues are not represented by linear peptides, (ii) posttranslational modifications are difficult to be recapitulated by synthetic peptides, and (iii) signal strength may be considerably lower with a single short peptide than with the full-length protein, in view that the autoimmune response in a given individual is usually polyclonal and several antigenic determinants are simultaneously recognized by different immunoglobulins. From these considerations, the use of full-length recombinant proteins, preferably expressed in mammalian cells to allow the typical posttranslational modifications, seems advantageous [42].

However, the use of full-length recombinant antigens brings other challenges. The purity of the recombinant proteins is of utmost importance for immobilization or direct labelling to achieve high specificity in the analytical assays. Detection of autoantibodies bound to immobilized full-length proteins can be reliably achieved by anti-immunoglobulin antibodies with suitable labels or tags for detection and signal amplification. Careful optimization of the coating process in such indirect ELISA tests, along with sufficient blocking of unspecific signals, are prerequisites for achieving the desired high signal to noise ratio [43]. The same challenge arises in the quantitative labelling of purified proteins, in order to avoid high background noise. While these approaches are straightforward and promising techniques when working with soluble proteins of limited size, it seems to be extremely difficult with large or even membrane proteins, as their purification is most challenging, costly, and only possible with some loss of material, protein quality, and integrity. A labor-intensive but reliable alternative is the generation of fusion proteins of the desired autoantigen with a sensitive reporter enzyme [44]. In this process, all recombinant antigens are reliably labeled by the reporter moiety exactly once, and no other protein that is co-purified during the preparation of the antigen receives the enzymatic label during assay set-up. In this way, the development of highly sensitive and specific assays seems possible, regardless of the size or subcellular localization of the antigen.

Several examples, where this approach has been successfully used for difficult-to-handle antigens, confirm this strategy. Human TSHR was expressed as a fusion protein with luciferase, allowing a highly sensitive and reliable assay for the diagnosis of TSHR-aAb in Graves’ disease [45]. The results obtained were in good agreement with the clinical features of the thyroid patients. However, the TSHR is a relatively atypical member of the GPCR family of endocrine receptors as it contains a very large N-terminal extracellular domain [29]. Other transmembrane proteins with only small portions extending into the extracellular space have also recently been targeted with this approach, including the two major thyroid iodide transporters, i.e., the sodium iodide symporter (NIS) and pendrin (PDS) [46]. Previous studies had shown that peptide-based assays gave incongruent results for NIS-aAb or PDS-aAb prevalence, ranging from 0–10% in controls, to 0–84% in Graves’ disease, and up to 0–97.5% in Hashimoto’s thyroiditis [46]. The results obtained with luciferase fusion proteins were better in line with the more sophisticated and elaborate assays that used radioactively labelled full-length transporters in precipitation assays, with prevalence of 1.8% to 5% in controls, 11.0–12.3% in Graves’ disease, and 4.7–7.5% in Hashimoto’s thyroiditis. The mandatory use of isotopes clearly posed a problem for the radioactive assays in terms of costs, general suitability in routine labs, and stability of the components used in the assays [47,48]. The development of similarly sensitive methods using recombinant full-length proteins fused to luciferase provides a promising alternative, in view of the equivalent results obtained by these two assay principles.

A similar discrepancy between peptide sequences and full-length proteins or biological assays also exists with other GPCR, e.g., in the field of cardiac receptors, where no consensus has been reached yet, as biological and peptide-based ELISA assays give very incongruent results [49,50]. The issue of excessive noise due to the nature of the antigens as GPCR questioned early findings that lacked appropriate negative and positive controls [51]. In PCOS, the hypothesis of a primary role for another centrally important GPCR, i.e., the GnRHR, was tested by different techniques [52,53]. The assays were based on an indirect measure of the biological activity of GnRHR-aAb [52], either on a peptide-based ELISA [52], or on a precipitation assay like in this study, quantifying the direct binding of GnRHR-aAb to the full-length receptor as luciferase fusion protein [53]. The results were incongruent, and an independent evaluation of these approaches is still required, but the latter study conducted by us questions the initial enthusiasm for this appealing hypothesis as specific autoantibodies binding the human GnRHR were observed in a tiny fraction of samples, only similar to the results of the present study. Notably, the positive samples identified in this study and presented here do not overlap to the positive patients in our former study on autoimmunity to the GnRHR [53].

The second important prerequisite for a sound analysis of our research hypothesis relates to the quality of the samples studied and the clinical characterization of the PCOS patients. Enrolment into the study and serum processing were performed in a standardized manner according to the highest principles of clinical research [54]. Blood collection and serum processing, as well as entry into the study database and as biological aliquots into the Graz Biobank, were performed strictly according to the agreed standard operating procedures as described [55]. Samples were sent on dry ice to the remote analytical laboratory in Berlin, Germany, and analyzed by personnel blinded to clinical characteristics. Samples were thawed and frozen only once prior to analysis, and serum quality tests routinely performed in Berlin using three different biomarkers for selenium status (total selenium, selenoprotein P concentration, and glutathione peroxidase-3 activity) yielded consistent results by spectroscopic, ELISA and enzyme activity assays, as described [56]. For these reasons, poor quality of samples, loss of immunoglobulins, or degradation of antibodies in the samples tested is not a likely explanation for the low number of positive samples identified. We are left with the notion that natural autoimmunity to the FSHR and LHR is a very rare event in PCOS, but may be of relevance in other conditions associated with subfertility or other hormonal imbalance.

The particular strengths of this study include the relatively large cohort sizes analyzed, the choice of detection principle, a successful parallel establishment of the analytical assays used, and the high quality of the clinical samples examined. Notable limitations include the lack of characterization of the FSHR-aAb assay by a commonly available antiserum (for our unsuccessful attempts to identify a suitable commercial product), the nature of the assays relying solely on binding without providing information on the potential biological activity of the aAb as agonistic, antagonistic, or merely neutral, and the lack of clinical information on the control samples other than sex, age and personal assessment as healthy. Moreover, only few positive samples were identified, and no consistent correlation to a relevant clinical parameter was observed due to the low frequency of positivity, rendering it impossible to determine relevant cut-offs for the binding index as measure of autoantibody concentrations in contrast to other similar studies [57]. Especially in view that the diagnosis of PCOS is difficult and often takes years to be done [58], we can moreover not exclude that the positive samples identified in the control cohort are just un-diagnosed cases of PCOS or display other signs of subfertility.

## 4. Materials and Methods

### 4.1. Human Samples

The cohort of PCOS patients consisted of n = 550 individual serum samples, collected in Graz, Austria, under highest quality standards, and stored at Biobank Graz [55]. The Rotterdam criteria for PCOS diagnosis were applied according to the literature [59,60]. Blood sampling was performed in the morning after an overnight fast. Standard anthropometric data, blood pressure, and other clinical parameters were measured as described [61]. All patients included had provided written informed consent prior to enrollment, and the investigation was conducted in accordance with the Declaration of Helsinki. An ethical approval had been issued by the review committee of Graz University (Ethikkommission der Medizinischen Universität Graz, Universitätsklinikum, Austria EC18-066 ex 06-07). Steroid hormones were determined in the samples routinely by ELISA (ADVIA Centaur^®^ Immunoassay, Siemens Healthcare Diagnostics Inc., Tarrytown, NY, USA), or liquid chromatography followed by mass spectrometry, as described earlier [53,62]. Control serum samples from subjects with a self-reported status as “healthy” (n = 400, 50% female) were obtained from a commercial supplier (In.Vent Diagnostica GmbH, Hennigsdorf, Germany). These samples were complemented by several control samples (female) from the biobank in Graz, collectively forming our cohort of controls.

### 4.2. Construction and Expression of GPCR-Luciferase Fusion Proteins

The fusion proteins of the two GPCR were constructed by the same principle as described before for the human GnRHR [53]. Briefly, the open reading frame of human FSHR was amplified by PCR using the forward primer FSHR-fwd (atggccctgctcctggtctctttg) in combination with FSHR-rev (atagaattcgttttgggctaaatgacttagagg), introducing an EcoR1 site (gaattc) in the reverse primer for allowing directed cloning into the expression vector. Similarly, the open reading frame of human LHR was amplified by using primers LHR-fwd (atgaagcagcggttctcggcgctg) and LHR-rev (atagaattcacactctgtgtagcgagtctt), also introducing an EcoR1 site (gaattc) in the reverse primer for enabling a directed insertion of the reading frame into the expression vector. Generation of the fusion proteins was achieved by assembling a reading frame encompassing the information for the GPCR coding sequence in front of a firefly luciferase cDNA. To this end, the reading frame of firefly luciferase was amplified with pSP-Luc+NF vector as template (Promega, Mannheim, Germany), as described [53]. The amplicon was inserted into the IRES-containing bicistronic vector pIRESneo (Addgene, Teddington, UK). The two newly generated expression vectors encoding for either FSHR-Luc or LHR-Luc (plasmid pIRESneo-FSHR-Luc) and plasmid pIRESneo-LHR-Luc, respectively) were sequence verified by a service provider (LGC Genomics GmbH, Berlin, Germany).

Recombinant expression of the fusion proteins was achieved in human embryonic kidney cells (HEK 293 cells). After transfection of plasmids using (FuGENE HD) reagent (Promega), cells were grown in DMEM containing 10% fetal bovine serum (FBS). Selection of clones having stably integrated the plasmid into their genomes was achieved by providing selection media containing the antibiotic geneticin (G418; 0.8 mg/mL, Sigma-Aldrich/Merck KGaA, Darmstadt, Germany). Clones expressing recombinant fusion proteins were selected by luciferase activity measurements and expanded in G418-containing media until reaching confluency. Aliquots of the stable clones were harvested and frozen to secure the most productive cell clones. Protein was prepared by scraping into PBS, centrifugation, and washing in PBS as described earlier [53]. Cell pellets were finally resuspended in 50 mM Tris, pH 7.4, 100 mM NaCl, 10% glycerol, and 10% Triton X-100 to lyse the cells and liberate soluble intracellular material. The membrane preparation was further washed, precipitated, resuspended, and aliquots were prepared and stored at −80 °C for the new analytical tests on autoimmunity to the human receptors.

### 4.3. Quantitative Analysis of FSH-R-aAb and LH-R-aAb in Human Serum Samples

The fusion protein preparations were diluted in 50 mM Tris, pH 7.5, 0.1 M NaCl, 10% glycerol, 5% glucose, 1% Triton X-100, and 5% milk powder (*w*/*v*), and activity of luciferase was determined as described [53]. Measurements were prepared on ice by providing 40 µL of diluted fusion protein in 96-well-plates. Serum samples were diluted with an equal volume of 50 mM Tris, pH 7.4, 100 mM NaCl, and 50% glycerol, and 10 µL of this dilution was used per measurement. Immune complexes between the luciferase fusion proteins and autoantibodies were allowed to form overnight at 4 °C and were bound to protein A (POROS-A, 10% (vol/vol), ASKA Biotech GmbH, Berlin) at room temperature and precipitated by centrifugation (500× *g*, 5 min, 4 °C). The pellets were washed three times with 50 mM Tris, pH 7.5, 100 mM NaCl, and 0.5% Triton X-100, and measured for luciferase activity in a luminometer (Mitras, Berthold Technologies GmbH, Bad Wildbad, Germany). The signals from precipitated autoantibody-fusion protein complexes were recorded as relative light units (RLU), using luciferin as substrate (Promega). A commercial antibody preparation (ThermoFischer Scientific, PA5-21271) for human LHR was identified and used to verify the LHR-aAb assay by dilution experiments.

### 4.4. Statistical Analyses

Statistical analyses were performed using GraphPad Prism v.9.1.2 (GraphPad Software Inc., San Diego, CA, USA). Relative binding indices (BI) were determined by building a ratio with the lower 50% of signals, assuming that more than half of the samples analyzed do not contain relevant amounts of GPCR-specific aAb. Hereby, the BI denotes the factor above background from negative samples. The threshold for positive autoimmunity was determined by applying the criterion for extreme mathematical outliers (P75 + 3xIQR).

## 5. Conclusions

We conclude that our newly generated diagnostic assays were of high quality and suitable to identify samples with detectable concentrations of FSHR-aAb or LHR-aAb. However, our results do not support the hypothesis of a relevant role of FSHR-aAb or LHR-aAb as a frequent cause of PCOS, indicating that other autoantigens may be targets of relevant autoimmunity in PCOS.

## Data Availability

The data presented in this study are available upon reasonable request from the corresponding author.
